# Evaluation of the effectiveness of using flipped classroom in puncture skills teaching

**DOI:** 10.1186/s12909-024-05132-8

**Published:** 2024-02-23

**Authors:** Weihao Zhang, Miao Jiang, Wei Zhao, Shuai Li, Fan Li, Feifei Feng, Yongjing Wang, Yan Li, Lan Liu

**Affiliations:** 1 Department of Gastroenterology, The Second Hospital of Shandong University, Jinan, Shandong 250033 China; 2 Clinical Skill Training Center, The Second Hospital of Shandong University, Jinan, Shandong 250033 China; 3 Department of Neurology, The Second Hospital of Shandong University, Jinan, Shandong 250033 China; 4 Department of Respiration, The Second Hospital of Shandong University, Jinan, Shandong 250033 China; 5 Department of Hematology, The Second Hospital of Shandong University, Jinan, Shandong 250033 China

**Keywords:** Flipped classroom, Traditional classroom, Medical students, Objective structured clinical examinations, Questionnaire

## Abstract

**Background:**

The effectiveness of flipped classroom (FC) on puncture skills in medical education is still uncertain. This study aimed to assess the role of the FC model in puncture skills and investigate the acceptance and approval of FC among medical students and instructors.

**Methods:**

A mixed research approach of quasi-experimental research design and descriptive qualitative research was conducted in September 2022 for one month, using an FC teaching method that combined instructional videos and group learning. The study participants were 71 fifth-year medical students from two classes at a Chinese medical school and four instructors. The medical students were randomly divided into two groups: the traditional classroom (TC) group (Group A) and the FC group (Group B). For teaching, Group B used FC, and Group A used PowerPoint-based TC. The effectiveness of the two teaching models was assessed with Objective Structured Clinical Examination (OSCE), and questionnaires were distributed to the medical students and instructors after the assessment. Two independent sample t-tests were used to analyse the differences in demographic data and the OSCE scores of the two groups of medical students.

**Results:**

Group B scored higher in puncture skills than Group A, especially regarding abdominal puncture (*p* = 0.03), thoracentesis (*p* < 0.001), bone marrow puncture (*p* < 0.001) and average performance of puncture skills (*p* < 0.001). For lumbar puncture, no difference in skill scores was observed between groups A and B (*p* > 0.409). The medical students thought that the FC improved their self-learning ability and helped them acquire knowledge. Regarding the OSCE of their skills, most medical students thought that it was more innovative and objective than traditional examinations and that it was better for assessing their overall abilities. Both the FC and OSCE were supported by the medical students. The instructors were also satisfied with the students’ performance in the FC and supported the teaching model, agreeing to continue using it.

**Conclusions:**

This study shows that FC teaching that combines instructional videos and group learning is a reliable and well-received teaching method for puncture skills, which supplements and expands existing teaching methods in the medical field.

**Supplementary Information:**

The online version contains supplementary material available at 10.1186/s12909-024-05132-8.

## Introduction

The COVID-19 pandemic has affected many sectors of medical education around the world, with many universities suspending on-campus teaching activities [1]. In early 2020, Chinese universities were actively teaching online, as required by the Chinese Ministry of Education. In this context, previous theoretical and practical teaching was considered no longer applicable, especially for practical skills such as internal medicine puncture skills. Therefore, it was necessary to adjust the medical teaching strategy as early as possible to ensure the smooth completion of the course. The flipped classroom (FC) is a blended learning model that combines lecture materials that are read or viewed prior to class with interactive face-to-face classrooms that actively engage students (The word ‘student’ refers to a ‘medical student’) [2]. This method addresses the problem of limited teaching time in the traditional lecture-based teaching model by allocating classroom time to the active application of the material that students learn before class [3]. However, most of the time, Chinese universities use the didactic model, which allows for a minimum number of instructors to convey information to a large number of students at the same time [4]. Medical students must master clinical procedures to be competent in a variety of clinical settings [5].

The Objective Structured Clinical Examination (OSCE) is a well-researched and proven method for assessing medical skills [6]; it comprehensively assesses a medical student’s ability to apply their medical knowledge and skills in clinical practice [7]. The OSCE uses multi-station assessment, standardised patients and virtual patients to assess candidates’ clinical skills in a fair and objective manner [8], and it is considered to be the most reliable clinical examination system in medical training [9]. In recent years, the OSCE has been widely used in China for the final assessment of residency training [10].

Although previous studies have shown that FC has a positive impact on several medical fields, it is still unknown whether FC can improve student performance in clinical skills [11], and few studies have evaluated the impact of FC on learning medical puncture skills. This study aimed (1) to assess the effectiveness of the FC in improving puncture skills performance by using a multi-component assessment and (2) to evaluate secondary endpoints such as student and instructors’ satisfaction with and acceptance of the FC and OSCE.

## Methods

A mixed research approach of quasi-experimental research design and descriptive qualitative research was conducted in September 2022 for one month as a pilot study at our institution. It was conducted in the Second Hospital of Shandong University and approved by the Ethics Committee of our hospital (LCLL-2022-011).

### Participants

All 71 medical students from two classes at a medical school and four instructors from medical school affiliates participated in this study. Inclusion criteria for the students included (1) voluntary participation in this study and signing of an informed consent form, (2) full-time undergraduate medical students and (3) no obvious physical or psychological abnormality. The exclusion criteria included having been exposed to flipped classroom teaching or having received training in puncture skills. The inclusion criteria for instructors included (1) having participated in the FC and TC teaching training organised and passing our hospital’s assessment; and (2) voluntarily participating in this study, complying with relevant regulations and signing of an informed consent form. The exclusion criteria included not participating in training related to this study or failing our hospital’s assessment.

### Study design

**Fig. 1:**
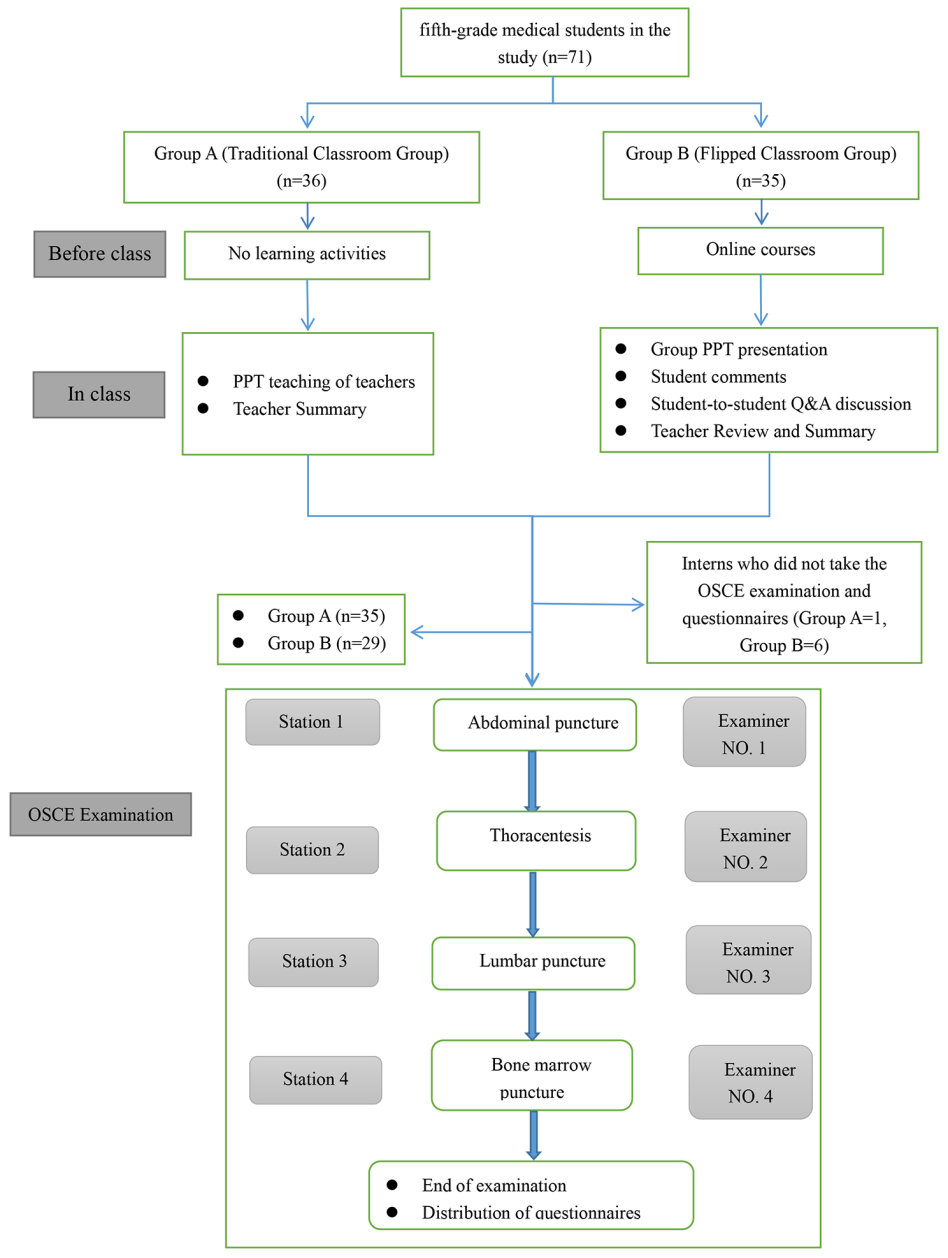
Study flowchart

This study adopted the FC teaching method, combining instructional videos with group learning. Before the study started, participants were randomly divided into groups A and B. Group B was taught using the FC teaching model and Group A using the traditional classroom (TC) teaching model. Randomisation and random assignment for this study was accomplished by the Random Number Table (URL: https://randomnumbergenerator.org/random-number-table). We numbered all students who volunteered for this study consecutively starting with 1 and then rearranged the serial numbers using a Random Number Table, with students coded ‘odd’ assigned to the traditional classroom (TC) group, and students coded ‘even’ assigned to the FC group. The flow chart of Fig. 1 shows the study design.

Group A did not have any learning task before class, and the instructors gave a face-to-face lecture for them using PowerPoint slides. Group B was randomly divided into four display groups, B1–B4, with random assignment, each of which was responsible for one puncture presentation in the form of PowerPoint presentations. Instructional videos on puncture skills were distributed to the members of Group B, who verbally agreed not to share the videos with Group A. A professional medical training institution produced the videos that were approximately 141 min long, of which 35, 40, 35 and 31 min were devoted to abdominal, thoracic, lumbar and bone marrow punctures, respectively. They outlined the purpose, indications, contraindications, introduction of operating items, demonstration of operation steps and explanation of operation problems of puncture. All puncture demonstrations were performed on body models. Before the lecture in the classroom, we asked group B to watch the instructional videos, and all Group B members completed this task. Group A were not given any self-study tasks, such as instructional videos, prior to their classroom instruction.

### Conceptual framework

In educational theory, Mayer’s cognitive multimedia learning system suggests that learning is most effective in an e-learning environment when both images and text are available [12]. Mayer argues that multimedia includes animation and narration, and his research involves using short multimedia tutorials [13], which considerably affect learning. Therefore, Mayer’s cognitive multimedia learning theory was used as a theoretical basis for this study.

The production of the lecture PowerPoint and the selection of instructional videos was based on the requirements of the notification document of the Chinese National Health Care Commission on the syllabus of the Physicians Qualifying Examination issued in 2019 (website: https://www.nmec.org.cn/Pages/ArticleInfo-13-11403.html). The theoretical objectives of the training courses include (1) indications and contraindications for puncture; (2) operational points; and (3) common problems with puncture and measures to solve them. The practical objective is to learn how to perform punctures on mannequins.

### Teaching process

The classroom courses for groups A and B were held separately once a week for four weeks. The sequence of lectures for both groups was abdominal puncture, thoracentesis, lumbar puncture and bone marrow puncture, and the duration of each session was controlled at 45 min.

Group A watched PowerPoints that were summarised by the lecturer, and the students asked questions. The instructors then summarised the important and more complex points of puncture skills. The classroom schedule of Group A was structured as follows: 30 min for the instructor’s PowerPoint presentation, 15 min for the students to ask the instructor questions, and 10 min for the instructor to summarise and comment. Group B used team-based learning [14], a group learning method, as follows: we randomly divided Group B into four groups of 7–8 people each using the random number table. The group (B1, B2, B3 or B4) in charge of the tasks for a particular week assigned two people to complete the PowerPoint presentation in the role of instructors. The other three groups commented on the PowerPoint presentation; then, they discussed the questions in the PowerPoint presentation in groups. The instructors briefly commented on errors and areas in the PowerPoint presentation that were difficult to understand. The classroom schedule of Group B was as follows: 25 min for group PowerPoint presentation, 10 min for group comments, 10 min for discussion between groups and 10 min for instructor’s comments and summary.

After the weekly lectures in the classroom, the two groups practiced the puncture skills in the Clinical Skills Training Center at the medical school, which provided the training location and equipment. Training time was limited to two hours, and the length, content and instructors for both groups A and B were the same.

### Main outcome measures

Groups A and B conducted the OSCE examination in the Clinical Skills Training Center of our hospital the day after they completed their skill training. The assessment items included four sites in the order of abdominal puncture, thoracentesis, lumbar puncture and bone marrow puncture. Each station was equipped with independent assessment space and equipment, including puncture simulator and puncture disinfection tools, and arranged for a professionally trained OSCE examiner to take charge of the examination. The preparatory work before the examination had been approved by our hospital’s OSCE Working Committee. The examiners used the OSCE assessment, and the process, content and examiners were the same for both groups. Figure 1 shows the OSCE assessment process.

### Secondary outcome measures

The questionnaires on the FC and the OSCE were designed by two of the study researchers and were administered in this study. The first two questionnaires focused on students’ acceptance and recognition of FC and OSCE; the other questionnaires aimed to evaluate instructors’ teaching habits and perceptions of the FC model. To assess the reliability of the questionnaires before the formal survey, 25 students were selected for the pre-assessment, and the results showed that the Cronbach’s α coefficients of the FC and OSCE questionnaires for students were 0.815, and their reliability met the requirements.

The evaluation dimensions of the FC questionnaire for students included pre-course and in-class perceptions of the content and methods of FC, acceptance, participation and suggestions for FC (additional file 1). The evaluation dimensions of the OSCE questionnaires included the assessment difficulties and effectiveness of OSCE, strengths and weaknesses, and recommendations (additional file 2). The FC questionnaire for instructors consists of open-ended questions, and its evaluation dimensions include teaching methods, evaluation of teaching effectiveness, levels of understanding of students, perceptions of FC teaching and development proposals. The survey gathered data from three perspectives: instructors’ past teaching experiences, teaching habits and knowledge of students’ skill acquisition and perceptions of the FC (additional file 3). In the FC and OSCE questionnaires for students, the Likert-scale answers to the questions ranged from 1 (*strongly disagree*) to 5 (*strongly agree*). At the end of the skills assessment, participants in both groups completed the questionnaires: Group A completed the OSCE questionnaire; Group B completed the OSCE and FC questionnaires for students, and the instructors completed the FC questionnaire for instructors.

### Statistical analysis

The normality and homogeneity of variance of the OSCE assessment data of groups A and B were analysed using IBM SPSS Statistics 26 (IBM Corp., Armonk, NY, USA). The data conforming to the normal distribution were described by the mean ± standard deviation, and the independent sample t-test was applied to analyse the differences between the two groups. The count data were described by the composition ratio (%); non-normally distributed data, described using rank means, were analysed with a non-parametric test (Mann-Whitney U). Statistical difference between the two groups was *p* < 0.05.

## Results

Table 1 shows the demographic characteristics of groups A and B. All 71 fifth-year medical students from two classes of the medical college and four instructors from a medical school-affiliated hospital participated in this study. The mean age of medical students in groups A and B was 27.60 ± 3.35 and 27.66 ± 2.99, respectively. The two groups showed no statistical differences in terms of gender (*p* = 0.866), age (*p* = 0.897) and marital status (*p* = 0.987), and none had previous experience of the FC teaching model.

**Table 1:** Demographic information

	Group				*P*
	A (*n* = 35)		B (*n* = 29)		
characteristics	Mean ± SD	N (%)	Mean ± SD	N (%)	
Age (year)	27.60 ± 3.35	/	27.66 ± 2.99	/	0.897
Gender (male/female)	/	9/26 (25.71%/74.29%)	/	8/21 (27.59%/72.41%)	0.866
Number of Married (N (%))	/	12 (34.29%)	/	10 (34.48%)	0.987
Experience in the FC	/	0	/	0	> 0.05

Age is expressed as mean ± standard deviation. Group B, flipped classroom; Group A, traditional classroom

### Results of skills assessment

Table 2 shows the results of the skills assessment for groups A and B. Thirty-five participants in Group A and 29 participants in Group B participated in the skills assessment. Group B had higher scores for abdominal puncture (*p* = 0.03), thoracentesis (*p* < 0.001) and bone marrow puncture (*p* < 0.001), and their average scores for puncture skills (*p* < 0.001) were higher than those of Group A. However, the two groups showed no statistical difference in lumbar puncture scores (*p* = 0.409).

**Table 2:** Skills assessment results

Assessment content	Group		*P*	Cohen’s d
	A (*n* = 35)	B (*n* = 29)		
Abdominal puncture	96.20 ± 1.41	97.00 ± 1.51	0.033*	0.548
Thoracentesis	96.23 ± 1.19	97.31 ± 1.11	< 0.001*	0.939
Lumbar puncture	96.03 ± 1.87	95.48 ± 3.30	0.409	0.205
Bone marrow puncture	93.66 ± 2.10	97.07 ± 1.36	< 0.001*	1.928
Average score of skills	95.53 ± 0.79	96.72 ± 1.06	< 0.001*	1.273

*It indicates a statistically significant difference between the scores of the two groups

### Results of questionnaires

A total of 29 valid FC questionnaires and 64 valid OSCE questionnaires were collected. One and six medical students in groups A and B, respectively, did not participate in the OSCE and complete the questionnaires. We investigated the reasons why students dropped out of the OSCE and questionnaires in groups A and B. One student in group A mentioned said that he had to take an elective exam on the day of the OSCE; four of the six students in group B also had to take an elective exam, while two were unable to do so for health reasons. None of the six students in group B dropped out of the OSCE and questionnaires because of the FC.

The four instructors completed the teaching-related FC questionnaires. The instructors involved in FC teaching were two men and two women, with an average age of 37 years. All of them held MD degrees and had an average teaching experience of 8.75 years. They all participated in FC teaching training for medical students, were well-versed in the FC model and passed our hospital’s FC teaching qualification examination.

### Questionnaires of students’ views of the OSCE

**Fig. 2:**
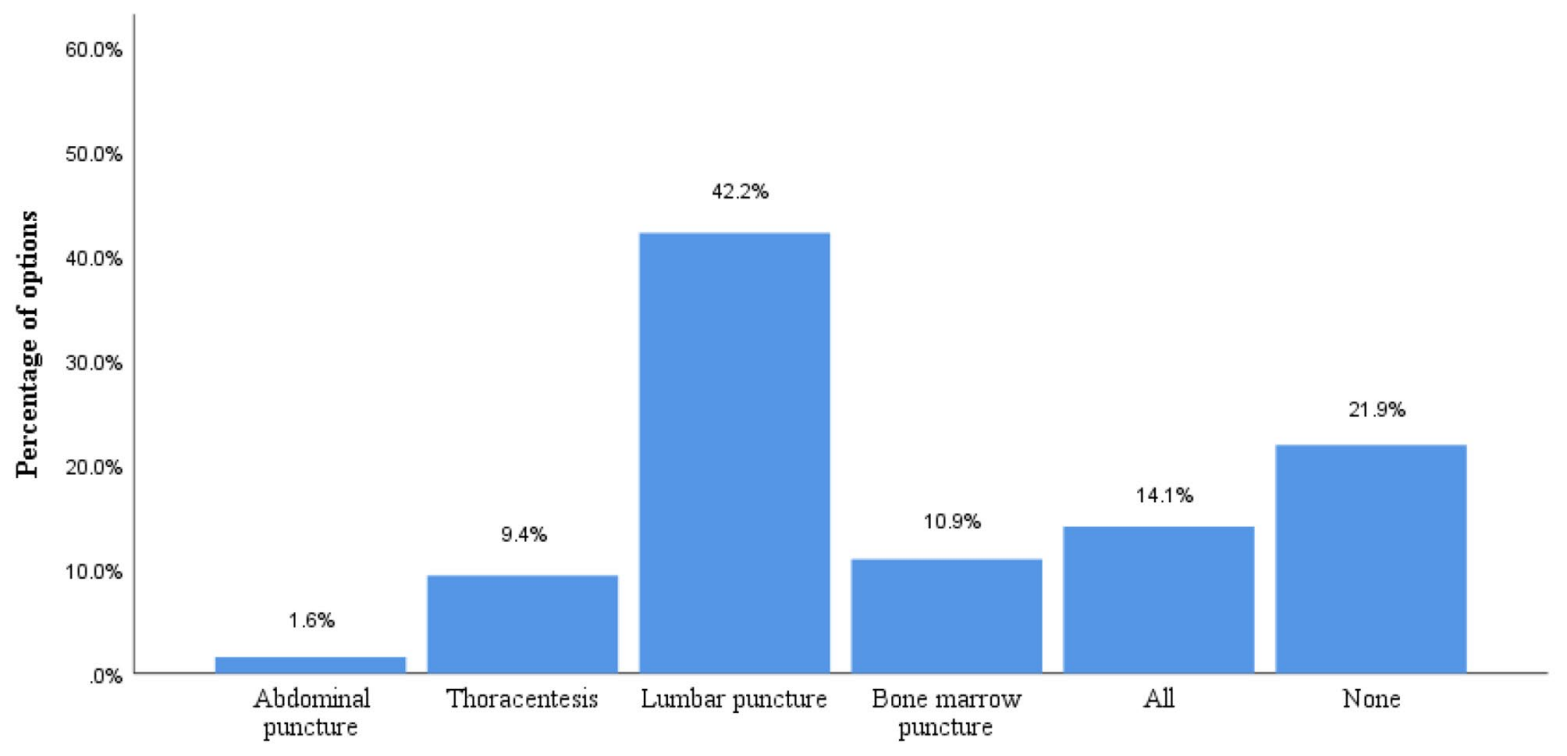
Which operation do you think is more difficult in this assessment? (Group A, B)

Regarding the difficulty of the puncture skills assessment, nearly half of the students thought that the lumbar puncture was the most challenging, followed by the bone marrow puncture, thoracentesis and abdominal puncture. One-fifth of the students thought that all four punctures were not difficult (Fig. 2). Regarding the OSCE assessment, most of the students said they understood it well (89.07%), that it truly reflected their competency levels (85.94%) and that this model helps medical students improve their overall competencies (90.63%). Compared to the traditional assessment model, more students said that the OSCE is more innovative and objective (92.19%), that they liked this type of skills assessment very much (85.94%) and that they agreed this type of assessment should be extended to the residency exam (78.12%; Table 3).

**Table 3:** FC, OSCE questionnaire results

Content	5	4	3	2	1
Q1: I understand the OSCE assessment model very well	30/64 (46.88%)	27/64 (42.19%)	7/64 (10.93%)	0	0
Q2: I believe that OSCE is beneficial to the overall competence of medical students in internship	36/64 (56.25%)	22/64 (34.38%)	6/64 (9.37%)	0	0
Q3: I believe that OSCE is a true reflection of the clinical competence of medical students	26/64 (40.63%)	29/64 (45.31%)	9/64 (14.06%)	0	0
Q4: OSCE is more innovative and objective than traditional exams	34/64 (53.13%)	25/64 (39.06%)	5/64 (7.81%)	0	0
Q5:I really like the way OSCE is evaluated	29/64 (45.31%)	26/64 (40.63%)	8/64 (12.50%)	0	1/64 (1.56%)
Q6: OSCE needs to be extended to the residency exam	26/64 (40.62%)	24/64 (37.50%)	11/64 (17.19%)	2/64 (3.13%)	1/64 (1.56%)
Q7: Through discussion and analysis in class, I have a deeper and more comprehensive grasp of knowledge	11/29 (37.93%)	17/29 (58.62%)	1/29 (3.45%)	0	0
Q8: Compared with TC, I think FC is more prominent in the improvement of students’ self-learning ability	10/29 (34.48%)	17/29 (58.62%)	2/29 (6.90%)	0	0
Q9: I would like our hospital to continue teaching in the FC model	10/29 (34.48%)	13/29 (44.83%)	5/29 (17.24%)	0	1/29 (3.45%)

FC: Flipped classroom; OSCE: Objective Structured Clinical Examination. Likert scores collected through a rating scale (5 = fully agree, 1 = fully disagree). Q1-Q6 are the contents of OSCE questionnaire, Q7-Q9 are the contents of FC questionnaire

### Questionnaires of students’ views of the FC

**Fig. 3:**
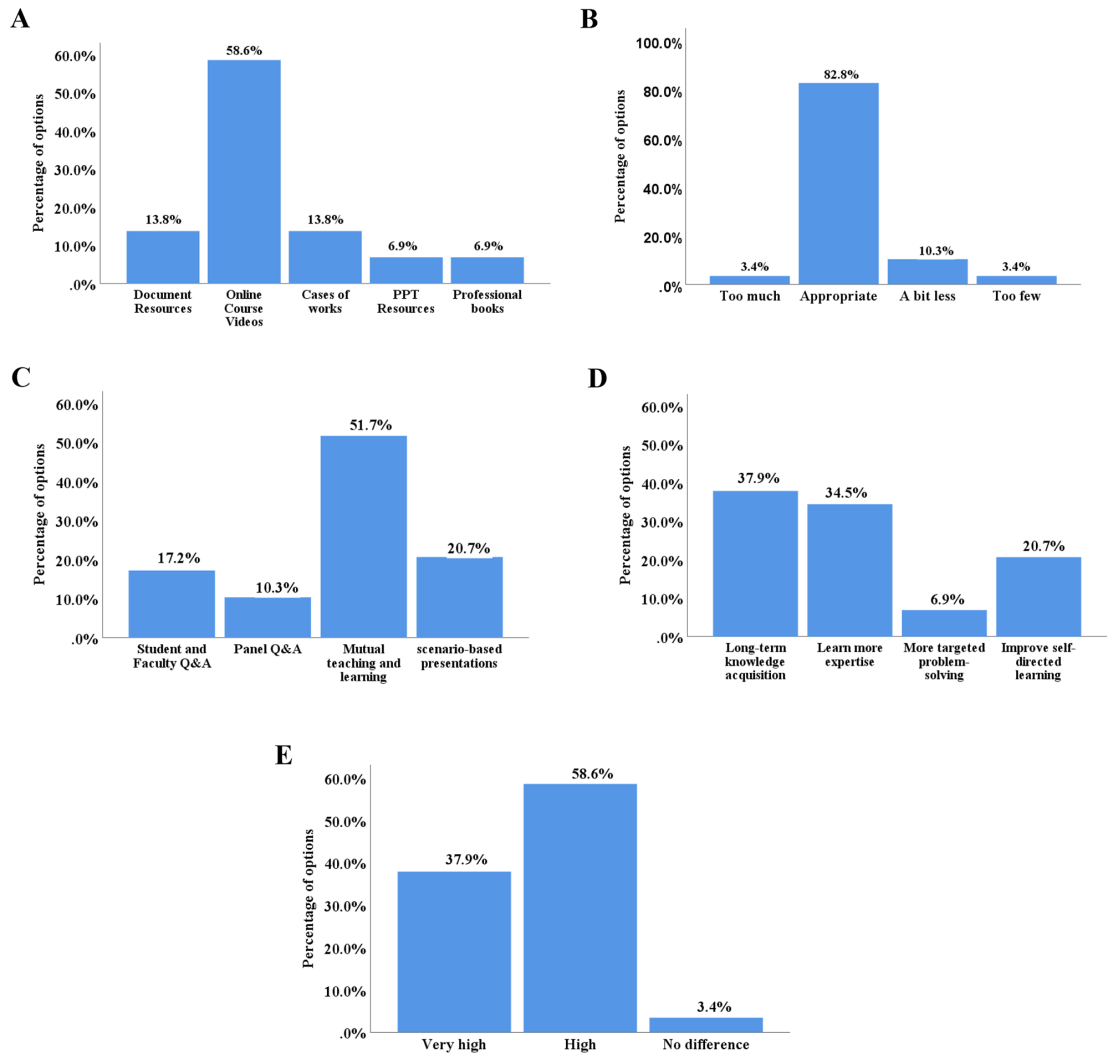
Survey of Group B interns’ views on FC. **A**: Which teaching resources do you think are the most suitable for your pre-course learning stage? **B**: Is the length of the learning resources provided in the pre-course period appropriate? **C**: Which do you think is the most effective way to interact and communicate in the classroom? **D**: What do you think about the learning effects of FC compared to TC? **E**: Compared with TC, what do you think is the learning efficiency of FC?

For the FC questionnaire, more than half of the students thought that the video lessons were the most appropriate way to learn about the procedures before class (Fig. 3A), and they thought the videos’ durations were appropriate (Fig. 3B). For the most effective way to interact in class, half of the students supported mutual teaching and learning, followed by scenario-based presentations, student and instructor Q&A, and student panel Q&A (Fig. 3C). Compared to the TC, one-third of the students thought the FC helped with long-term knowledge acquisition; another third said the FC provides more specialised knowledge (Fig. 3D), and more than half of the students thought the FC was more effective than the TC (Fig. 3E). Most of the students thought that the analysis and discussion of problems in the FC led to a more comprehensive and deeper mastery of knowledge (96.55%); they thought that the FC was more effective for improving self-learning skills than the TC (93.10%), and they supported the hospital in continuing to promote the FC teaching model (79.31%; Table 3).

### Questionnaires of instructors’ views of the FC

The FC questionnaires for instructors showed that all instructors were well aware of the FC model and had had appropriate teaching experience before the study. They all preferred the FC model to TC, were satisfied with FC’s effectiveness and the medical students’ performance, supported the FC model and agreed to continue using it. When we asked the instructors about the preference of these two teaching methods (teaching effectiveness and student performance satisfaction), they all said that they preferred FC and believed that TC’s teaching effectiveness and student performance satisfaction are worse than those of FC.

## Discussion

During the COVID-19 global pandemic, instructors around the world have been using FC to teach in a fully online environment. However, the teaching of operational skills such as puncture skills is facing challenges with regard to students (not familiar with FC, difficult to accept online courses), instructors (increased workload of lesson preparation) and operational training [15]. To meet this challenge during the epidemic teaching period (COVID-19) and provide a realistic basis for the development of FC in medical teaching in China, this study assessed the FC model’s value, which combines instructional videos with group learning in teaching puncture skills to medical students.

Our research showed that using the FC model led to more highly skilled performance of abdominal, thoracic, lumbar and bone marrow puncture when compared to the TC among fifth-year medical students. We surveyed the students’ perceptions of the FC and OSCE after they took their puncture skills examination, and they generally had a high level of recognition and acceptance of the FC and the OSCE, supporting their promotion in future clinical teaching and assessment. This study is similar to the one by Sana, which stated that a blended teaching approach based on video learning and simulation teaching improved students’ OSCE scores and performance compared to TC [16]. Similarly, a meta-analysis conducted by Hew et al. showed that medical students responded well to video learning and interactive discussions in class and stated that this teaching method helped improve learning motivation and better the understanding of learning topics [17]. Our study demonstrates the positive effect of the FC model, which combines instructional videos with group learning, on medical students learning clinical skills in an environment with limited clinical teaching resources.

### Analysis of puncture skills performance

Studies from several medical schools have shown that medical students have inadequate exposure to basic clinical procedures during their studies, and they feel no confidence performing them [18, 19]. This finding highlights the need for a more effective approach to ensuring adequate learning and practice opportunities in clinical skills teaching [20]. Many healthcare institutions worldwide have significantly increased the number of students to address the shortage of healthcare workers, which greatly reduces opportunities for learning practical skills [21]. Research has shown that watching instructional videos is one way to implement a FC and can be a viable response to increased student numbers [22]. Combining videos with face-to-face instruction can improve medical students’ knowledge and their performance of clinical skills [20]. The FC allows students to learn the knowledge independently before class by watching videos or using other learning media; thus, class time is freed up for them to apply their knowledge and actively participate in higher-level thinking [23].

In our study, Group B had higher scores in puncture skills scores compared to Group A, which suggests that the FC teaching model improves puncture skills– a finding also confirmed by studies of other categories of clinical skills [24–26]. However, groups A and B (TC and FC) showed no difference in scores for the lumbar puncture, and the mean score was the lowest of the four punctures overall. The questionnaire showed that nearly half of the students thought lumbar puncture was the most difficult of the four punctures skills, which is consistent with the results of existing studies [19, 27]. We hypothesised that the operational difficulty of the lumbar puncture affects students’ learning and assessment self-confidence, which in turn affects the final operational outcome [23]. Some studies have shown that teaching and practicing with lumbar puncture simulators can increase students’ self-confidence [28, 29]. A recent study on asthma teaching similarly compared the effects of FC and TC on medical students’ test scores [30]. Although they found that FC did not improve test scores, most of the participants were satisfied with FC and stated that it improved their motivation to learn. All of the above illustrated the positive impact of FC on medical students’ learning.

### Analysis of the results of the questionnaires about students’ views on FC

After the assessment, students in Group B answered a questionnaire survey on the FC. Regarding the pre-course learning method, most students thought that the videos were appropriate. Studies showed that video resource learning was equivalent to bedside teaching and lectures for clinical skills training [22, 31]. Although the total duration of the puncture videos was nearly 2.5 h, we learned through the questionnaire that this length was acceptable to most of the students. This finding is quite different from previous studies, which have shown that most students prefer short videos [32, 33]. Different preferences for video length might be determined by students’ learning motivation [34]. The puncture in our research involved more steps than the other punctures; therefore, a longer video demonstration was needed, and understandably, students were receptive to this longer video.

When answering the question about the most effective way to communicate and interact in the classroom, half of the students in Group B supported teaching each other. The FC comprises student-centred classroom learning activities that ultimately lead to learning interest and a greater focus on the task or tasks being learned [13]. We also surveyed students’ acceptance of the FC. The results of the questionnaire showed that most of the students supported the continuation of the FC model, indicating that the FC had a very high acceptance level among the students. Medical students face the dual pressures of academics and clinical practice daily, and the FC allows them the flexibility to develop their learning plans without being restricted to a particular time and place [35].

### Analysis of the questionnaire results about students’ views on the OSCE

The OSCE has gained worldwide popularity for its comprehensiveness and objectivity in evaluating medical students’ clinical competence [36, 37]. In this study, we assessed the puncture skills of the interns with the OSCE. The questionnaire showed that most of the students were well aware of this exam model, and they had a high level of acceptance and approval of it; however, a small number of students felt that it increased their stress levels and affected their performance. One study showed that the OSCE is one of the most anxiety-producing assessments for learners [38]. Improving examiner training, reducing the time between the exam and getting feedback on the results, and allowing students to fully understand the feedback may be one strategy for reducing immediate anxiety [39].

### Analysis of the questionnaire results about instructors’ views on FC

Based on the questionnaire responses, we found that the FC was supported by the instructors– a result consistent with previous studies [11, 40]. A study of FC among ophthalmic trainees found that teachers were more satisfied with FC in teaching ocular trauma traineeships compared to TCs, and it met their teaching expectations. Moreover, the study speculated that the inclusive, lively, and student-centred nature of the FC model may have contributed to its popularity [11]. However, in our study, some instructors reported that the FC requires a great deal of preparation, including the production of pre-class instructional videos and interactive content for class. These instructors also face heavy clinical and research pressure, and often, they do not have enough energy to conduct the FC. One study noted that instructors can reduce the stress of teaching by utilizing existing teaching resources or collaborative lesson planning [41]. Therefore, it is necessary to continue studying FC methods in clinical teaching work and adapt them to the characteristics of different clinical disciplines, optimize the teaching structure and reduce the teaching pressure on instructors.

The innovations of this study are several. In terms of research methods, this study creatively utilized a mixed research approach, combining a quasi-experimental research design with descriptive qualitative research. This approach is an improvement over previous one-sided research methods as it evaluates the skills and teaching experience of both instructors and students using quantitative and qualitative methods. This study highlights that the FC model not only improves students’ performance in puncture skills assessment, but it also increases students’ and instructors’ acceptance of and satisfaction with puncture skills courses. In this research design, the study integrates the FC teaching method, which combines instructional videos with collaborative learning for the first time. This teaching method can assess the effectiveness and benefits of FC through two processes of pre-class preparation and classroom teaching, resulting in a more scientific and comprehensive evaluation. This also indicates that the TC has some issues, such as limited inclusiveness and abstract teaching content.

Our study showed that FC combining the instructional videos with group learning performed comparable or even better than TC in improving the assessment performance of medical students’ puncture skills; the recognition and acceptance of the FC teaching model by students and instructors were high, and they supported its further promotion for clinical skills teaching. Our study complements the current research on applying FC in the teaching of puncture skills. During the current disease pandemic, FC deserves further promotion in the medical field.

### Limitations

Our study had several limitations. First, it was monocentric: although students were receptive to the content and length of the pre-course learning videos, their views do not necessarily represent the opinions of students in other clinical teaching centres. Second, although students completed pre-course video learning, we do not have detailed data on individual viewing habits and how much they comprehended these puncture skills. Finally, as the difference in abdominal, thoracentesis and bone marrow skills in this study is not substantial, increasing the sample size may make it more significant. This result also shows that the FC model has the same or even better effectiveness on puncture skill performance than TC. It is worth noting that four of the six dropouts in Group B did not participate in the OSCE and questionnaires because they had to take the elective examinations, and the other two could not participate in the OSCE and questionnaires due to health reasons. Thus, the absence of the above six records is completely random and will not be biased against the final results.

## Conclusions

This study supplements and expands the existing teaching methods in the medical field by addressing the learning effectiveness of FC, combining instructional videos with group learning on the puncture skills of medical students. Future research could expand this teaching method to other clinical disciplines according to their characteristics.

### Electronic supplementary material

Below is the link to the electronic supplementary material.

Supplementary Material 1

Supplementary Material 2

Supplementary Material 3

## Data Availability

The datasets used and analysed in this study are available from the corresponding authors upon reasonable request.

## Abbreviations

FC: Flipped classroom
OSCE: Objective Structured Clinical Examination
TC: Traditional classroom
