# Supplementary material for: Evaluation of the effectiveness of using flipped classroom in puncture skills teaching
Source: BMC Med Educ. 2024 Feb 23;24:176. doi: 10.1186/s12909-024-05132-8 (PMC10885647; doi:10.1186/s12909-024-05132-8)
Supplement: Supplementary file 1 — Supplementary Material 1 [file 12909_2024_5132_MOESM1_ESM.doc]

Additional file 1. Flipped classroom Questionnaires for students

| Dear medical students: | | | | | | | | |
| --- | --- | --- | --- | --- | --- | --- | --- | --- |
| Thank you very much for taking the time to participate in this survey. The purpose of this survey is to investigate your perceptions of the flipped classroom model after it has been implemented. We hope to provide a basis for this research. The survey will take some time and your responses will be used for this survey only. This survey is completely anonymous and privacy is guaranteed. Thank you again for your support. | | | | | | | | |
| 1. Name. | | | | | | | | |
| 1. Age and marital status. | | | | | | | | |
| 1. Which teaching resources do you think are the most suitable for your pre-course learning stage? | | | | | | | | |
| 1. Document resources | | 1. Online courses videos | | 1. Cases of works | | 1. PPT resources | | E.Professional books |
| 1. Is the length of the learning resources provided in the pre-course period appropriate? | | | | | | | | |
| 1. Too much | 1. A little more | | 1. Appropriate | | 1. A bit less | | 1. Too few | |
| 1. Through discussion and analysis in class, I have a deeper and more comprehensive grasp of knowledge. | | | | | | | | |
| 1. Strongly agree | 1. Agree | | 1. Neutral | | 1. Disagree | | 1. Strongly disagree | |
| 1. Which do you think is the most effective way to interact and communicate in the classroom? | | | | | | | | |
| 1. Students and Faculty Q&A | 1. Panel Q&A | | 1. Mutual teaching and learning | | 1. Scenario-based presentation | | 1. Team Competition | |
| 1. Compared with traditional classroom, I think FC is more prominent in the improvement of students' self-learning ability. | | | | | | | | |
| 1. Strongly agree | 1. Agree | | C. Neutral | | D. Disagree | | E. Strongly disagree | |
| 1. What do you think about the learning effects of FC compared to TC? | | | | | | | | |
| 1. Long-term knowledge acquisition | 1. Learn more expertise | | 1. More targeted problem solving | | 1. Improve self-directed learning | | 1. All the same | |
| 1. Compared with TC, what do you think is the learning efficiency of FC? | | | | | | | | |
| 1. Very efficient | 1. Highly efficient | | 1. General efficiency | | 1. Less efficient | | 1. Very inefficient | |
| 1. I would like our hospital to continue teaching in the FC. | | | | | | | | |
| 1. Strongly agree | B. Agree | | C. Neutral | | D. Disagree | | E. Strongly disagree | |
| 1. Give your opinion or suggestion about FC. | | | | | | | | |

FC: flipped classroom; TC: traditional classroom.
