# Supplementary material for: Evaluation of the effectiveness of using flipped classroom in puncture skills teaching
Source: BMC Med Educ. 2024 Feb 23;24:176. doi: 10.1186/s12909-024-05132-8 (PMC10885647; doi:10.1186/s12909-024-05132-8)
Supplement: Supplementary file 2 — Supplementary Material 2 [file 12909_2024_5132_MOESM2_ESM.doc]

Additional file 2. Objective Structured Clinical Examination (OSCE) Questionnaires for students

| Dear medical students: | | | | | | | | | |
| --- | --- | --- | --- | --- | --- | --- | --- | --- | --- |
| Thank you very much for taking the time to participate in this survey. The purpose of this survey is to investigate your perceptions of the OSCE assessment model after it has been conducted. We hope to provide a basis for this study, and the survey will take some time. your responses will be used for this survey only. This survey is completely anonymous and privacy is guaranteed. Thank you again for your support. | | | | | | | | | |
| 1. Name. | | | | | | | | | |
| 1. Age and marital status. | | | | | | | | | |
| 1. Which operation do you think is more difficult in this assessment? | | | | | | | | | |
| 1. Abdominal | 1. Thoracentesis | | 1. Lumbar puncture | | 1. Bone marrow puncture | | | 1. All | 1. None |
| 1. I understand the OSCE assessment model very well. | | | | | | | | | |
| 1. Strongly agree | | 1. Agree | | 1. Neutral | | 1. Disagree | 1. Strongly disagree | | |
| 1. I believe that OSCE is beneficial to the overall competence of medical students in internship. | | | | | | | | | |
| 1. I believe that OSCE is a true reflection of the clinical competence of medical students. | | | | | | | | | |
| 1. Strongly agree | | 1. Agree | | 1. Neutral | | 1. Disagree | 1. Strongly disagree | | |
| 1. OSCE is more innovative and objective than traditional exams. | | | | | | | | | |
| 1. Strongly agree | | 1. Agree | | 1. Neutral | | 1. Disagree | 1. Strongly disagree | | |
| 1. I really like the way OSCE is evaluated. | | | | | | | | | |
| 1. Strongly agree | | 1. Agree | | 1. Neutral | | 1. Disagree | 1. Strongly disagree | | |
| 1. OSCE needs to be extended to the residency exam. | | | | | | | | | |
| 1. Strongly agree | | 1. Agree | | 1. Neutral | | 1. Disagree | 1. Strongly disagree | | |
| 1. Provide your views or suggestions on the OSCE evaluation model. | | | | | | | | | |
