# Supplementary material for: Evaluation of the effectiveness of using flipped classroom in puncture skills teaching
Source: BMC Med Educ. 2024 Feb 23;24:176. doi: 10.1186/s12909-024-05132-8 (PMC10885647; doi:10.1186/s12909-024-05132-8)
Supplement: Supplementary file 3 — Supplementary Material 3 [file 12909_2024_5132_MOESM3_ESM.doc]

Additional file 3. Flipped classroom questionnaires for instructors

| Dear instructor: | | | |
| --- | --- | --- | --- |
| Thank you very much for taking the time to participate in this survey. The purpose of this survey is to investigate your perceptions of the flipped classroom model after its implementation and to hopefully provide a basis for this research. The survey will take up some of your time, and your responses will be used for the purpose of this survey only. Thank you again for your support. | | | |
| 1. Gender. | | | |
| 1. Name, age and marital status. | | | |
| 1. Subjects and teaching experience. | | | |
| 1. What kind of medical students have you taught in the past? | | | |
| 1. Undergraduate medical trainees | 1. Undergraduate Medical Internship | 1. Internal Medicine Residency | 1. Other types (please fill in specifically) |
| 1. Have you attended any formal training courses before to improve your teaching skills? | | | |
| 1. Yes | | 1. No | |
| 1. If you answered "yes" above, please describe the training course content specifically. | | | |
| 1. [Fill in the blank] Regarding the pre-classroom situation of the FC, please give your personal opinion in terms of how much you knew about the FC, how much you knew about the learners' skill level, and whether you asked the learners to familiarize themselves with the steps of skills before the class. | | | |
| Please fill in the box with your answer: | | | |
| 1. [Fill in the blanks] Regarding the situation during the lessons, please give your personal opinion on whether the learning objectives of the lesson were clearly and concisely stated to the learners, whether the expected level of performance of the skills taught in the lesson was stated, whether the steps for performing the skills were explained, and whether the correct and incorrect practices were clearly explained to the learners. | | | |
| Please fill in the box with your answer: | | | |
| 9. [Fill in the blanks] Regarding the post-course practice of piercing skills, please give your personal opinion on whether the learners were given sufficient time to practice their skills and whether they were given specific feedback on operational problems and suggestions for improvement. | | | |
| Please fill in the box with your answer: | | | |
| 10. [Fill in the blanks] Regarding the perception of learners' learning ability and the evaluation of the quality of the lecture, please give your personal opinion on whether the limitations of learners were recognized and whether the focus of this lecture was outstanding. | | | |
| Please fill in the box with your answer: | | | |
| 11. [Fill in the blank] Regarding the two teaching methods: FC and TC, please give your personal opinions on how much you favor these two teaching methods, the effectiveness of the teaching, and the satisfaction of students' classroom performance through these two teaching methods. | | | |
| Please fill in the box with your answer: | | | |
| 1. [Fill in the blank] Please give your personal opinion on whether you would like our hospital to continue the FC teaching pilot. | | | |
| Please fill in the box with your answer: | | | |

FC: flipped classroom; TC: traditional classroom.
